# Dry Needling at Myofascial Trigger Spots of Rabbit Skeletal Muscles Modulates the Biochemicals Associated with Pain, Inflammation, and Hypoxia

**DOI:** 10.1155/2012/342165

**Published:** 2012-12-23

**Authors:** Yueh-Ling Hsieh, Shun-An Yang, Chen-Chia Yang, Li-Wei Chou

**Affiliations:** ^1^Department of Physical Therapy, Graduate Institute of Rehabilitation Science, China Medical University, Taichung 40402, Taiwan; ^2^Department of Physical Medicine and Rehabilitation, Tzu Chi General Hospital, No. 66 Sec. 1 Fongsing Road, Tanzih Township, Taichung 42743, Taiwan; ^3^Department of Physical Medicine and Rehabilitation, China Medical University Hospital, No. 2 Yuh-Der Road Taichung 40447, Taiwan; ^4^School of Chinese Medicine, College of Chinese Medicine, China Medical University, Taichung 40402, Taiwan

## Abstract

*Background and Purpose*. Dry needling is an effective therapy for the treatment of pain associated with myofascial trigger point (MTrP). However, the biochemical effects of dry needling that are associated with pain, inflammation, and hypoxia are unclear. This study investigated the activities of *β*-endorphin, substance P, TNF-*α*, COX-2, HIF-1*α*, iNOS, and VEGF after different dosages of dry needling at the myofascial trigger spots (MTrSs) of a skeletal muscle in rabbit. Materials and Methods. Dry needling was performed either with one dosage (1D) or five dosages (5D) into the biceps femoris with MTrSs in New Zealand rabbits. Biceps femoris, serum, and dorsal root ganglion (DRG) were sampled immediately and 5 d after dry needling for *β*-endorphin, substance P, TNF-*α*, COX-2, HIF-1*α*, iNOS, and VEGF immunoassays. *Results*. The 1D treatment enhanced the *β*-endorphin levels in the biceps femoris and serum and reduced substance P in the biceps femoris and DRG. The 5D treatment reversed these effects and was accompanied by increase of TNF-*α*, COX-2, HIF-1*α*, iNOS, and VEGF production in the biceps femoris. Moreover, the higher levels of these biochemicals were still maintained 5 d after treatment. *Conclusion*. Dry needling at the MTrSs modulates various biochemicals associated with pain, inflammation, and hypoxia in a dose-dependent manner.

## 1. Introduction

Myofascial pain syndrome (MPS) is characterized by an acute or chronic regional muscle pain primarily caused by myofascial trigger points (MTrPs) located in taut muscle bands, fascia, or tendinous insertions [1–7]. The diagnosis of MPS is based on the identification of MTrPs in the taut band by palpation of tender nodules, local twitch response, and specific pain referral patterns associated with each MTrP [3, 7, 8]. 

To date, the neurophysiology and pathogenesis of MPS and MTrP have not been confirmed. Local compression by taut bands (muscle fibers that shorten in the absence of propagating action potentials) can impair arterial inflow and reduce the supply of oxygen, calcium, and other nutrients necessary for energy-dependent muscle relaxation and higher energy demands required by the aberrantly sustained muscle contraction [7]. Continued, persistent sarcomere contractures can distort and damage involved tissues, which may precipitate the synthesis and release of endogenous algogenic biochemicals and inflammatory substances that enhance nociception [3, 4, 9]. These effects suggest that persistent or chronic pain perception associated with MPS can involve numerous proinflammatory cytokines, neurotransmitters, and neuromodulators, including tumor necrosis factor-*α* (TNF-*α*), substance P, and cyclooxygenase-2 (COX-2), which relay pain signals from the peripheral to the central nervous system. An increase in *β*-endorphin level can suppress neurons from releasing substance P and thus, inhibit pain transmission [10]. These observations, which provide biochemical evidence, correlated with recent, partially approved studies that demonstrated significantly higher concentrations of substance P and TNF-*α* in the local milieu of active MTrPs [9, 11]. Moreover, a number of hypoxic-responsive proteins, including hypoxia-inducible factor-1*α*, vascular endothelial growth factor (VEGF), and inducible isoform of nitric oxide synthases (iNOS), can be found in response to hypoxia and mechanical stimulation in skeletal muscles [12, 13].

Dry needling (i.e., without injectate) at MTrPs is an effective therapy that inactivates an MTrP if performed in a particular manner [14–18]. A systematic review of MPS treatment found that dry needling is as effective as lidocaine injections [18, 19]. However, other comparative studies reported on the negative effects of dry needling on the management of MTrPs [20]. These inconsistencies may have resulted from limited sample sizes, poor quality of placebo-controlled trials, and protocol (technique, particularly in eliciting local twitch responses (LTRs); dosage; and duration) of dry needling [4, 15, 18–20]. Therefore, the biochemical responses underlying the antinociceptive effects and pain relief associated with dry needling must be clarified to develop adequate treatment measures for MPS. To achieve this goal, the fluctuating levels of *β*-endorphin, substance P, TNF-*α*, COX-2, HIF-1*α*, iNOS, and VEGF at different dosages of dry needling at the myofascial trigger spots (MTrSs, similar to human MTrPs) of a skeletal muscle were assessed in the present study through a well-established rabbit model.

## 2. Materials and Methods

### 2.1. General Design

**Figure 1:**
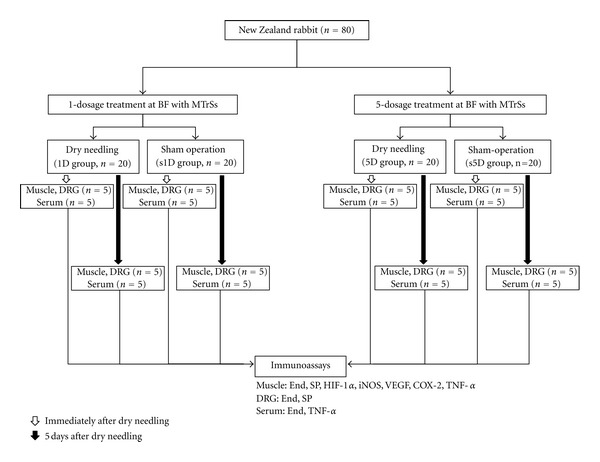
Flow chart for the animal study. Abbreviations: 1D, one-dosage dry needling; 5D, five-dosage dry needling; BF, biceps femoris; COX-2, cyclooxygenase-2; DRG, dorsal root ganglion; End, *β*-endorphin; HIF-1*α*, hypoxia-inducible factor-1*α*; iNOS, inducible isoform of nitric oxide synthases; MTrS, myofascial trigger spot; SP, substance P; TNF-*α*, tumor necrosis factor-*α*; VEGF, vascular endothelial growth factor; s1D, sham one-dosage dry needling; s5D, sham five-dosage dry needling.

The fluctuating levels of biochemicals after dry needling to the rabbit biceps femoris containing MTrSs were examined. The MTrS of the biceps femoris muscle on a randomly selected side was treated with predetermined dosages of dry needling. A total of 80 rabbits were randomly and equally divided into four groups based on the dosages of dry needling: (1) the one-dosage dry needling (1D) group (*n* = 20) that received one session of dry needling, (2) the one dosage of sham dry needling (s1D) group (*n* = 20) that received one session of sham dry needling, (3) the five-dosage dry needling (5D) group (*n* = 20) that received five daily sessions of dry needling for five consecutive days, and (4) the five dosages of sham dry needling (s5D) group (*n* = 20) that received five daily session of sham dry needling. Half of the animals in each group were sacrificed on the day immediately after dry needling, and the remaining animals were sacrificed 5 d after dry needling for immunoassays and serology test (Figure 1). The immunoassays include the following: (1) *β*-endorphin, substance P, TNF-*α*, COX-2, HIF-1*α*, iNOS, and VEGF in the bicep femoris muscles containing MTrSs, (2) *β*-endorphin and substance P in the dorsal root ganglion (DRG) corresponding to the biceps femoris, and (3) TNF-*α* and *β*-endorphin in the serum. Individual serological tests for assessing serum *β*-endorphin and TNF-*α* levels were sampled before and after dry needling in five randomly selected animals from each group to avoid the effects of repetitive drawing on the results of immunoassays in the muscle and DRG. The flow diagram is presented in Figure 1.

### 2.2. Animal Care

Adult male New Zealand rabbits (ages 16 weeks to 20 weeks, body weight between 2.5 and 3.0 kg) were used in the experiments. The animals were housed individually in standard polycarbonate tub cages lined with a wood chip bedding and had unlimited access to food and water. The cages were placed in an air-conditioned room (25°C ± 1°C), with 40 dBA noise, and a 12 h alternating light-dark cycle (6:00 a.m. to 6:00 p.m.). Each animal was housed and cared for according to the ethical guidelines of the International Association for the Study of Pain in animals [21, 22]. Effort was made to minimize discomfort and reduce the number of animals used. All animal experiments were conducted by following the procedure approved by the Animal Care and Use Committee of a university in accordance with the Guidelines for Animal Experimentation. The general experimental conditions were essentially the same as those previously described [23–25]. 

### 2.3. Animal Model for a Myofascial Trigger Point Study

Hong and Torigoe [26] developed an animal model for an MTrP study on rabbits [26]. A specific hyperirritable spot (MTrS) in the rabbit biceps femoris muscle is similar to the human MTrP. LTRs can be elicited at this spot when the needle tip encounters a sensitive locus. Spontaneous electrical activities, including endplate noise and endplate spikes, can also be recorded frequently within this sensitive spot, similar to a human MTrP [27, 28]. This animal model has been used in numerous studies on myofascial pain [23–25, 27, 29, 30] and thus was deemed appropriate for the current study. 

### 2.4. Identification of MTrS

Prior to administration of an anesthetic, the tenderest spots (i.e., MTrS) of the bicep femoris were identified by a finger pinch. The animal's reaction was observed (withdrawal of the lower limb, turning the head, screaming, etc.) to confirm the exact location of an MTrS. These painful regions were marked on the skin with an indelible marker. The animals were then anesthetized with 2% isoflurane (AErrane, Baxter Healthcare of Puerto Rico, PR, USA) in the oxygen flow for induction, followed by a 0.5% maintenance dose [31]. The body temperature was monitored by placing the thermistor probe of a thermometer (Physitemp Instruments, Inc., Clifton, NJ, USA) in the rectum. The temperature was maintained at approximately 37.5°C by using a body temperature control system consisting of a thermostatically regulated DC current heating pad and an infrared lamp. The biceps femoris of a marked hindlimb was grasped from behind the muscle, and the muscle was palpated by gently rubbing (rolling) it between the fingers to find a taut band. A taut band has the texture of a clearly delineated “rope” of muscle fibers and is roughly 2 mm to 3 mm or more in diameter. This area was designated for the dry needling treatment.

### 2.5. Dry Needling of the Biceps Femoris Muscle

All needling procedures were performed by the same investigator who was blinded to the group assignment regarding the needling dosage. Dry needling stimulation was performed with a disposable 30G acupuncture needle (300 *μ*m in diameter, 1.5 in length, Yu-Kuang Industrial Co., Ltd., Taiwan). The technique used was similar to the other methods used in our previous studies [25, 32]. In needling the MTrS of the biceps femoris, the needle was first inserted perpendicularly through the skin at the center of the marked spot. The needle was then advanced slowly and gently into the muscle until the needle tip touched the bone surface to estimate the thickness of the muscle. Needle rotation was simultaneously performed to facilitate a fast “in-and-out” needle movement, eliciting as many LTRs as possible. Sham needling was performed by inserting the needle into the subcutaneous layer of the marked MTrS region at a depth of approximately 1 mm to 2 mm from the skin surface. The needle remained inserted without any further movement.

### 2.6. Serum and Tissue Preparations

Blood (1 mL) was drawn from the marginal ear vein of the rabbit under light anesthesia (4% isoflurane) before and after treatments for five rabbits from each group. Blood samples were collected on chilled glass tubes and then left in an ice-filled bucket for 3 h to 4 h at −80°C until the day of the experiment. After the treatment, the rest of the animals were immediately sacrificed (i.e., without having blood samples drawn from them) with strong anesthesia by injecting saturated KCl (300 g/mL, intraperitoneal injection) to harvest the biceps femoris muscle containing the taut band and its ipsilateral corresponding segment of L2–L5 DRG for the immunoassays. The muscle specimens were cut through the midline and were then divided into two portions to obtain identical specimens for immunohistochemical staining and immunoassays. For immunohistochemical staining, the muscle specimens were fixed in 10% neutral formalin and then embedded in paraffin for 12 h at room temperature. For the immunoassays, the specimens were homogenized in T-PER Tissue Protein Extraction Reagent (Pierce Chemical Co., IL, USA) and the complete cocktail of protease inhibitors (Sigma, NY, USA). After centrifugation, the supernate was extracted and stored at −80°C. The specimen biceps femoris muscles with nontaut bands were also harvested from some rabbits. The specimens were then submitted for hematoxylin and eosin (H&E) staining to identify and compare the morphology of taut and nontaut bands within a muscle. 

### 2.7. Enzyme-Linked Immunosorbent Assay (ELISA)

The levels of serum *β*-endorphin and TNF-*α* were determined by ELISA (*β*-endorphin: Catalog no. E0806Rb, EIAab Science Co., Ltd., Wuhan, China; TNF-*α*: DuoSet ELISA Development kit R&D Systems, Minneapolis, MN, USA). Specimen extracts were incubated in 96-well plates coated with an antibody specific to *β*-endorphin or TNF-*α*. Biotin-conjugated *β*-endorphin or TNF-*α* and horseradish peroxidase-conjugated streptavidin (HRP) were added and incubated according to manufacturer's instructions. After washing, a tetramethylbenzidine substrate solution was added. The enzyme reaction was terminated by adding a stop solution containing sulfuric acid. The concentration of *β*-endorphin in serum and TNF-*α* in the biceps femoris was assessed with a reader (Thermo Scientific Multiskan EX, Finland) using a 450 nm filter and normalized with an abundance of the standard solution. Data were then analyzed using Ascent Software (Thermo Scientific Ascent Software, Finland) and a four-parameter logistic curve fit. 

### 2.8. Western Blot Analysis

Protein determination was performed using modified Lowry protein assays. Equal amounts of protein were loaded and separated in 10% Tris-Tricine SDS-PAGE gels. The resolved proteins were then transferred onto polyvinylidene fluoride membranes (Millipore, Bedford, MA, USA). The membranes were blocked in 5% nonfat milk for 1 h at room temperature and then incubated overnight with primary antibodies against *β*-endorphin (Cat. # ab8907, Abcam, Cambridge, UK), substance P (Cat. # orb11399, Biorbyt, Cambridge, UK), HIF-1*α* (NB100-105, Novus Biologicals, CA, USA), VEGF (Cat. # 205907, Abbiotec, CA, USA), iNOS (Cat. # AB5382, Millipore, CA. USA), COX-2 (Cat. # 250609, Abbiotec, CA, USA), and glyceraldehyde 3-phosphate dehydrogenase (GAPDH) (ab8245, Abcam Inc, MA, USA) at a dilution of 1 : 2500 in a blocking solution. The blots were then incubated with the HRP-conjugated goat anti-mouse and anti-rabbit anti-Immunoglobulin G (IgG) secondary antibody (1 : 20000, Jackson ImmunoResearch Laboratories, Inc., West Grove, PA, USA) for 1 h at room temperature. The signals were finally visualized using an enhanced chemiluminescence detection system (Fujifilm LAS-3000 Imager, Tokyo, Japan), and the blots were exposed to X-ray. All Western blot analyses were performed at least three times, and consistent results were obtained. The immunoreactive bands were analyzed using computer-based densitometry Gel-Pro Analyzer Version 6.0 (Media Cybernetics, Inc. USA). The gray levels obtained from the densitometric analysis of the immunoreactive bands were normalized with GAPDH (14C10, Cell Signaling Technology, Danvers, MA, USA).

### 2.9. Immunohistochemical Staining and Quantitative Analysis

The specimens were subjected to xylene diaphanisation, dehydrated using graded ethanol, embedded in paraffin, and then sliced using a microtome into 4 *μ*m thick cross sections. Each muscle specimen produced approximately 20 sections. For immunohistochemical staining, the slides were first incubated overnight at 4°C with a monoclonal mouse anti-TNF-*α* antibody (1 : 200, ab1793, Abcam, MA, USA). The muscle sections were then incubated with biotinylated goat anti-mouse IgG secondary antibody (Jackson ImmunoResearch Laboratories, Inc., West Grove, PA, USA) for 1 h at room temperature. After washing the sections, they were incubated with a streptavidin-HRP conjugate (Jackson ImmunoResearch Laboratories, Inc., West Grove, PA, USA). The sections were visualized as brown precipitates by adding 0.2 mg/mL 3,3′-diaminobenzidine (DAB) (Pierce, Rockford, IL, USA) as a substrate. The sections were then counterstained with hematoxylin. The immunohistochemically stained sections were examined under a light microscope (BX43, Olympus America Inc., NY, USA). 

The slides were examined and photographed at five randomly selected fields at 200x magnification under a light microscope (BX43, Olympus America Inc., NY, USA) and with a 1360 pixels × 1024 pixels cooled digital color camera (DP70, Olympus America Inc., NY, USA). The images were saved and adjusted to equalize contrast and brightness by using Adobe Photoshop (CS3, San Jose, CA); no other modifications were made. The digital images were analyzed by computer-based morphometry using ImageScope software package with the Color Deconvolution v9 tool (v9.1.19.1571, Aperio, Vista, CA, USA). Based on the automatically calculated parameters, the number of DAB-stained strong-positive staining cells in each section was measured for TNF-*α*-like immunoreactivity (TNF-LI).

### 2.10. Statistical Analysis

Experiments were repeated at least five times for each sample. The relative intensity of the Western blot band for each protein was normalized to the GAPDH protein level, expressed as a percentage of its sham value. The data are expressed as mean ± standard deviation. The differences in the contents of *β*-endorphin, substance P, TNF-*α*, COX-2, HIF-1*α*, iNOS, and VEGF among the four groups were determined by ANOVA. Scheffe's method was used to perform post hoc comparisons among groups. A *P* value of <0.05 was considered statistically significant. All data were analyzed using SPSS version 10.0 for Windows (SPSS Inc., IL, USA).

## 3. Results

### 3.1. Histopathological Evaluation of the Biceps Femoris with a Taut Band and a Nontaut Band

**Figure 2:**
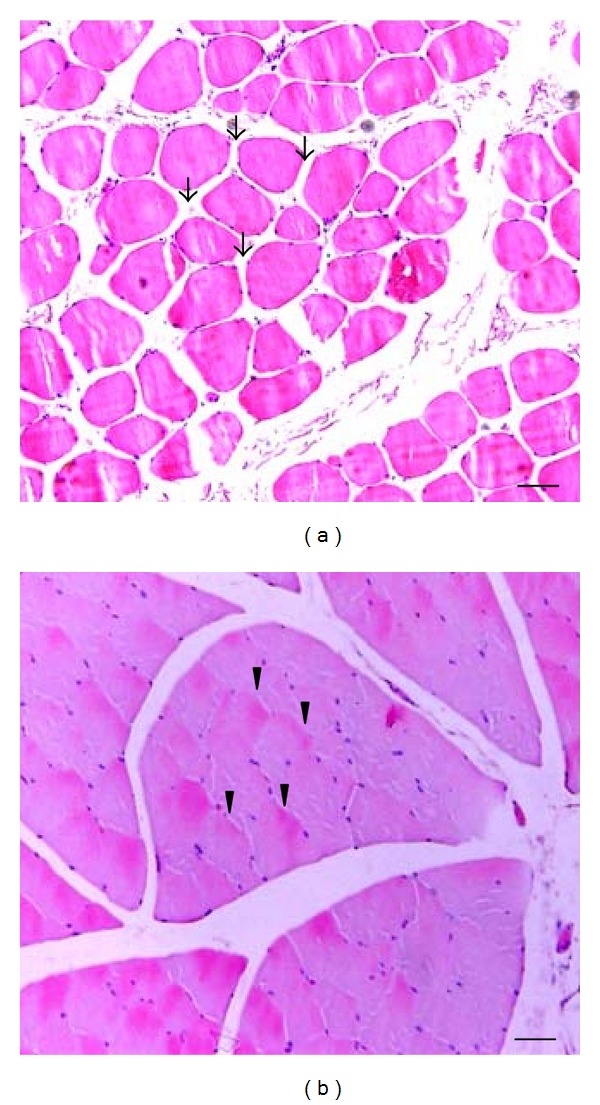
Morphological findings of representative skeletal muscles with nontaut and taut bands. (a) Biceps femoris with a nontaut band; (b) Biceps femoris with a taut band (H&E staining, scale bar = 5 *μ*m).

In the histomorphometric analysis by H&E staining, the nontaut band portion of the biceps femoris muscle showed a skeletal muscles with normal morphology, characterized by polygonal fibers with multiple nuclei arranged on the periphery of the cell and a clear space of endomysium enveloping each muscle fiber (Figure 2(a)). However, the endomysium enveloping each muscle fiber in the taut band of the biceps femoris was narrow and cramped, suggesting an enlargement of muscle fibers due to shortening and tightness of the contractile unit or focal muscle edema (Figure 2(b)). 

### 3.2. Effects of Dry Needling on the Protein Levels of *β*-Endorphin in the Biceps Femoris and Serum

**Figure 3:**
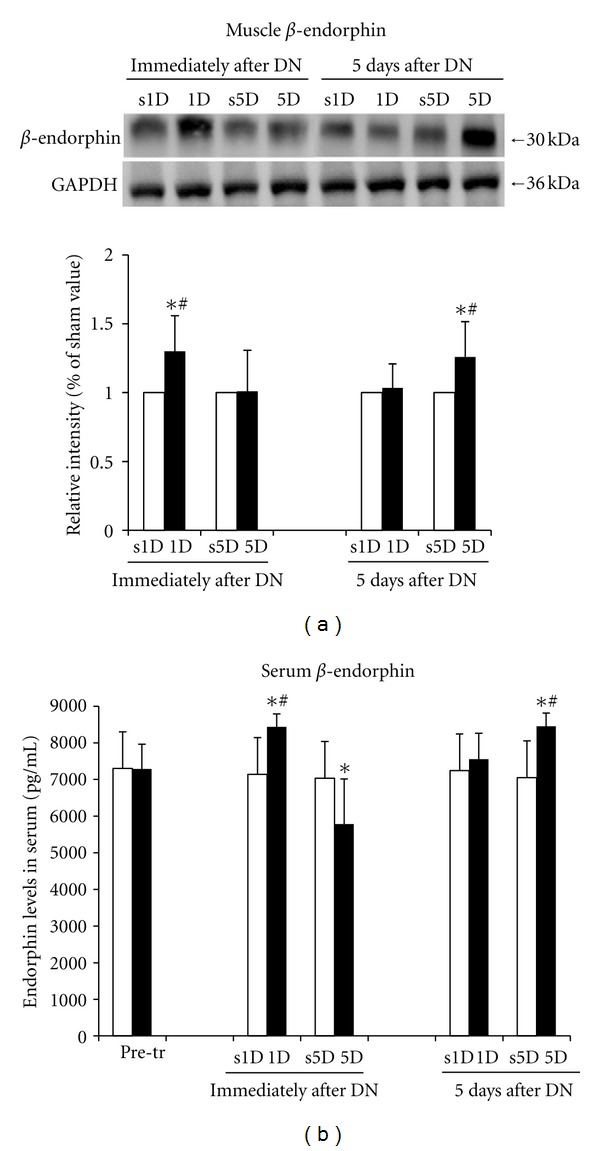
Effects of one- and five-dosage (1D, 5D) dry needling on *β*-endorphin expression in the needling-treated muscle and serum. The levels of *β*-endorphin in the muscle and serum obtained by (a) Western blot analysis and (b) ELISA. A representative Western blot image of a muscle tissue is shown (upper panel of (a)). The quantification of the protein levels is expressed as mean ± SD. *Indicates a significant difference (*P* < 0.05) between the sham groups (s1D and s5D). ^#^Represents the significant difference (*P* < 0.05) between the 1D and 5D groups. DN: dry needling.

The protein levels of *β*-endorphin in the biceps femoris for the 1D, s1D, 5D, and s5D groups are presented in Figure 3(a). Immediately after treatment, the protein levels of *β*-endorphin in the needling-treated biceps femoris significantly increased in the 1D group compared with the s1D group (*P* < 0.05). No significant difference in the protein levels of *β*-endorphin was indicated between the 5D and s5D groups (*P* > 0.05). However, 5 d after dry needling, the *β*-endorphin levels in the biceps femoris significantly increased in the 5D group (*P* < 0.05) and remained the same in the 1D group (*P* > 0.05). 

The protein levels of serum *β*-endorphin determined by ELISA for the 1D, s1D, 5D, and s5D groups are shown in Figure 3(b). No significant difference in the serum *β*-endorphin levels between the s1D and s5D groups was indicated before, immediately after, and 5d after the treatment (all *P* > 0.05). Serum *β*-endorphin significantly increased immediately after the 1D treatment (*P* < 0.05) but significantly decreased immediately after the 5D treatment (*P* < 0.05). Five days after dry needling, serum *β*-endorphin significantly increased in the 5D group (*P* < 0.05) but not significantly changed in the 1D group (*P* > 0.05). Significant differences between the 1D and 5D groups were observed immediately and 5 d after treatment (all *P* < 0.05).

Significant differences in the *β*-endorphin levels in the biceps femoris and the DRG were determined between the 1D and 5D groups immediately and 5 d after treatment (all *P* < 0.05). The biceps femoris and serum in the 1D group showed higher levels of *β*-endorphin compared with the 5D group immediately after treatment (all *P* < 0.05). However, these levels were lower in the 5D group than in the 1D group 5 d after treatment (all *P* < 0.05).

### 3.3. Effects of Dry Needling on the Protein Levels of Substance P in the Biceps Femoris and the DRG

**Figure 4:**
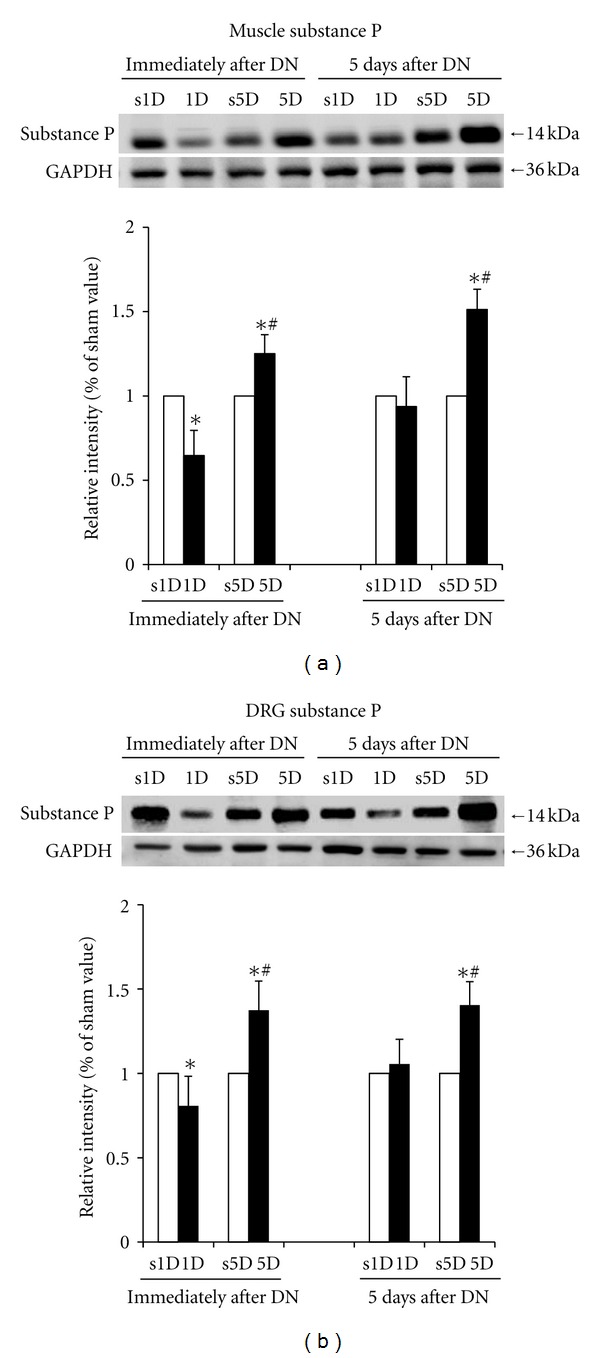
Effects of one- and five-dosage dry needling (1D, 5D) on substance P expression in a (a) needling-treated muscle and (b) DRG. Representative Western blot images are presented on the upper panels of (a) and (b). The quantification of the protein levels is expressed as mean ± SD. *Indicates the significant difference (*P* < 0.05) between the sham groups (s1D and s5D). ^#^Represents the significant difference (*P* < 0.05) between the 1D and 5D groups. DN: dry needling.

The protein levels of substance P in the biceps femoris of the 1D, s1D, 5D, and s5D groups are shown in Figure 4(a). The protein levels of substance P in the needling-treated biceps femoris significantly decreased in the 1D group compared with the s1D group immediately after treatment (*P* < 0.05). However, immediately after 5D dry needling, the substance P protein in the biceps femoris significantly increased compared with the s5D group (*P* > 0.05). Five days after dry needling, no significant differences in the protein levels of substance P were observed between the 1D and s1D groups (*P* > 0.05). However, a prolonged increase in substance P in the biceps femoris was observed in the 5D group compared with those in the s5D group (*P* < 0.05).

The protein levels of substance P in L2–L5 DRG with the corresponding innervations of the biceps femoris for the 1D, s1D, 5D, and s5D groups are shown in Figure 4(b). These results were similar to the protein levels of substance P in the biceps femoris. 

Significant differences in substance P of the biceps femoris and DRG were observed between the 1D and 5D groups at the time points immediately and 5 d after treatment (all *P* < 0.05). Higher levels of substance P in the biceps femoris and the DRG were found in the 5D group compared with those in the 1D group at the time points immediately and 5 d after treatment (all *P* < 0.05).

### 3.4. Effect of Dry Needling on the Protein Levels of iNOS, HIF-1*α*, COX-2, and VEGF in the Biceps Femoris

**Figure 5:**
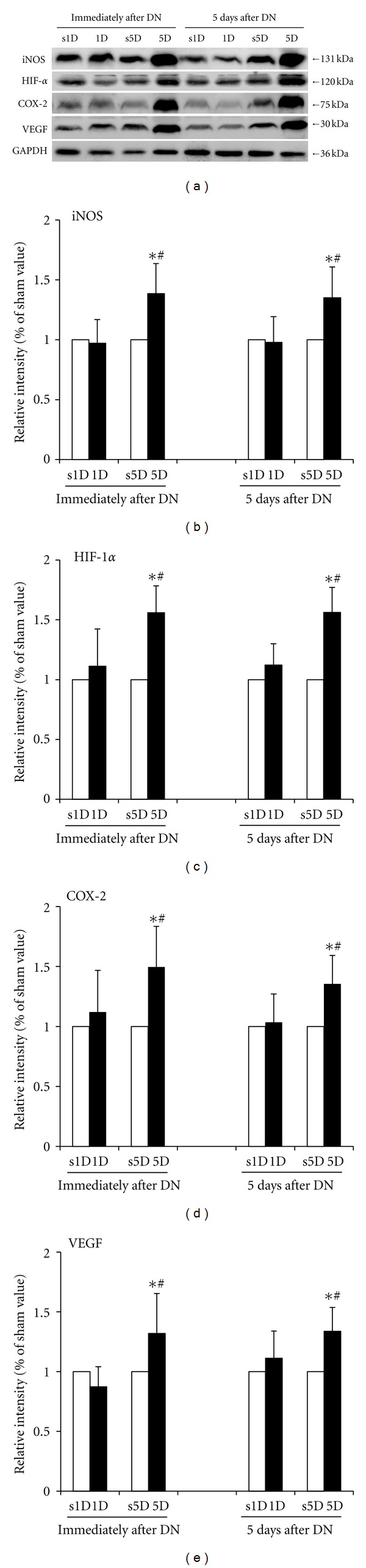
Effects of one- and five-dosage dry needling (1D, 5D) on iNOS, HIF-1*α*, COX-2, and VEGF expressions in a needling-treated muscle. (a) Representative Western blot images. The quantification of the protein levels for (b) iNOS, (c) HIF-1*α*, (d) COX-2, and (e) VEGF. Values are expressed as mean ± SD. *Indicates the significant difference (*P* < 0.05) between the sham groups (s1D and s5D). ^#^Represents the significant difference (*P* < 0.05) between the 1D and 5D groups. DN: dry needling.

The protein levels of iNOS, HIF-1*α*, COX-2, and VEGF in the biceps femoris for the 1D, s1D, 5D, and s5D groups are shown in Figure 5. No significant differences in the protein levels of iNOS, HIF-1*α*, COX-2, and VEGF were observed between the 1D and s1D groups at the time points immediately and 5 d after dry needling (*P* > 0.05). However, after the 5D dry needling, the levels of iNOS, HIF-1*α*, COX-2, and VEGF proteins were significantly higher than those in the s5D group (all *P* < 0.05). The increase was prolonged and observed 5 d after the 5D dry needling (all *P* < 0.05). 

Significant differences in the protein levels of iNOS, HIF-1*α*, COX-2, and VEGF between the 1D and 5D groups were indicated at the time points immediately and 5 d after treatment (all *P* < 0.05). Higher levels of iNOS, HIF-1*α*, COX-2, and VEGF in the biceps femoris were observed in the 5D group compared with those in the 1D group at the time points immediately and 5 d after treatment (all *P* < 0.05).

### 3.5. Immunoassays for TNF-*α* in the Biceps Femoris and Serum after Dry Needling

**Figure 6:**
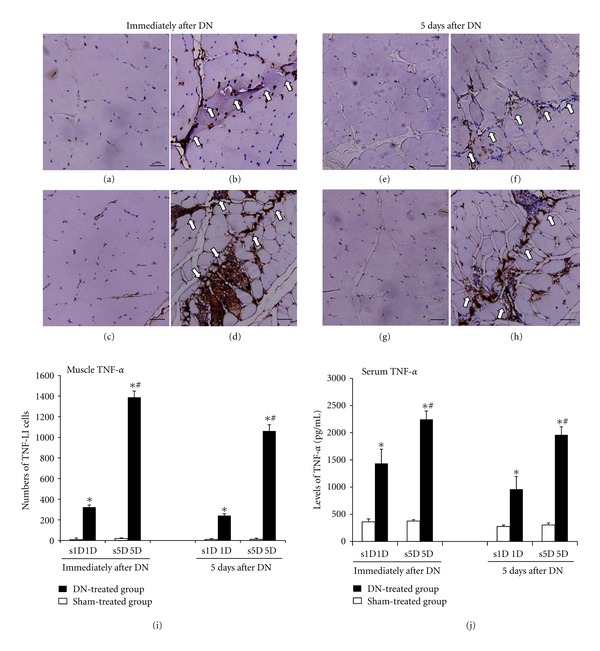
The variation in TNF-*α* after one- and five-dosage dry needling (1D, 5D) in the biceps femoris with a taut band in the experimental (1D, 5D) and sham groups (s1D, s5D). Representative photomicrographs indicate the immunohistochemical labeling for TNF-*α* in the muscle immediately after dry needling: (a) s1D, (b) 1D, (c) s5D, and (d) 5D, as well as 5 d after dry needling: (e) s1D, (f) 1D, (g) s5D, and (h) 5D. Histograms indicate the quantitative analysis of (i) TNF-LI cells and (j) protein levels of TNF-*α* by applying ELISA in the serum. *Indicates the significant difference (*P* < 0.05) between the sham groups (s1D and s5D). ^#^Represents the significant difference (*P* < 0.05) between the 1D and 5D groups. DN: dry needling. (scale bar = 5 *μ*m).

Immunohistochemical analysis of the 1D and 5D groups showed that the biceps femoris sections of the rabbits exhibited marked inflammatory cell infiltration and more TNF-LI cells along the needling path compared with the s1D and s5D groups immediately after dry needling (all *P* < 0.05; Figures 6(a), 6(b), 6(c), 6(d), and 6(i)). Five days after dry needling, both 1D and 5D groups continued showing marked inflammation along the needling path compared with the s1D and s5D groups (all *P* < 0.05; Figures 6(e), 6(f), 6(g), and 6(h)). The needling-treated muscle in the 1D group almost healed with a slight inflammatory cell accumulation. In the 5D group, the muscle had not fully healed 5 d after dry needling, with some inflammatory cells and TNF-LI cells clustered in the needling area. Scarring in the needling area was evident. In the 5D group, the inflammatory and TNF-LI cells significantly increased and were overexpressed compared with those in the 1D group immediately and 5 d after dry needling (all *P* < 0.05, Figure 6(i)). In the s1D and s5D control groups, the muscle fibers exhibited regularly arranged fascicles without degeneration, hemorrhage, necrosis, inflammatory cell infiltration, or TNF-LI cell accumulation. The protein levels of TNF-*α* in serum assessed by ELISA also increased significantly in the 1D and 5D groups compared with those in the s1D and s5D groups (all *P* < 0.05, Figure 6(j)). These results obtained by ELISA were similar to those obtained by immunohistochemistry. 

Significant differences were indicated in the TNF-*α* levels of the biceps femoris and serum between the 1D and 5D groups immediately and 5 d after treatment (all *P* < 0.05). Higher levels of TNF-*α* in the biceps femoris and serum were observed in the 5D group compared with those in the 1D group immediately and 5 d after treatment (all *P* < 0.05). 

## 4. Discussion

Table 1 summarizes the main results of the study. This study is the first to report on assessing biochemical alterations after dry needling MTrSs in a well-established animal model. Our findings suggested differences in the dry needling-modulated biochemicals associated with pain, inflammation, and hypoxia between the 1D and 5D treatments. These variations are dosagedependent and based on the levels of substance P and *β*-endorphin, as well as those of TNF-*α*, iNOS, HIF-1*α*, COX-2, and VEGF.

### 4.1. Short-Term Dry Needling Modulates the Biochemicals Associated with Pain and Inflammation

In this study, substance P, *β*-endorphin, and TNF-*α* were responsive to a short-term (1D) dry needling treatment. The effects on these biochemicals associated with pain and inflammation showed the following: (1) immediately after the 1D treatment, an increase in the TNF-*α* levels in the biceps femoris and the *β*-endorphin levels in the biceps femoris and serum was accompanied by a reduction in the substance P levels of the biceps femoris and DRG; and (2) 5 d after the 1D treatment, these variations in the substance P and *β*-endorphin levels were not observed, but TNF-*α* continued to accumulate along the needling path in the biceps femoris through which the needle was manipulated in and out.

Peripheral opioid analgesia has received considerable attention as an endogenous pathway of inhibiting pain. Studies showed that increasing the *β*-endorphin level in the inflamed tissues can cause analgesia [33, 34]. Acupuncture was demonstrated to enhance the secretion of endogenous opioid and *β*-endorphin in blood plasma to produce a strong analgesic effect and control pain in peripheral tissues [35]. Opioids are anti-inflammatory because they inhibit the release of neuroinflammatory mediators, including substance P [10]. An electroacupuncture treatment can reduce the mechanical allodynia in a mouse model of cancer pain because of a consequent decrease in substance P levels in the spinal dorsal horn and an increase in *β*-endorphin levels in the blood and the brain [36]. Shah et al. found elevated levels of substance P in subjects with an active MTrP in the upper trapezius muscle during needle insertion. The elicitation of an LTR resulted in a significant decrease in substance P concentration [9, 11]. Our result showing the decrease in dry needling-evoked substance P was consistent with that in the study by Shah et al. [9, 11]. The data obtained from this study suggested that the 1D treatment produces a short-term analgesic effect by modulating the substance P and *β*-endorphin levels in peripheral sites; however, no lasting effect was observed 5 d after dry needling.

A systematic review showed marked improvements in patients with MPS in which MTrPs were directly needled, suggesting that dry needling therapy has a specific efficacy in the treatment of pain arising from MTrPs [18]. However, some clinical trials demonstrated that dry needling achieves only short-term alleviation of pain and improvement of function [37, 38]. Our biochemical findings described above suggest that one mechanism by which short-term dry needling produces brief analgesia in MPS may be the enhancement of peripheral *β*-endorphin in the serum and the biceps femoris. 

### 4.2. Long-Term Dry Needling Modulates the Biochemicals Associated with Pain, Inflammation, and Hypoxia

In addition to alterations of substance P, *β*-endorphin, and TNF-*α* caused by the 1D treatment, COX-2, HIF-1*α*, iNOS, and VEGF were more responsive to long-term (5D) dry needling treatment. For these biochemicals associated with pain, inflammation, and hypoxia, the following was observed: (1) immediately after the 5D treatment, TNF-*α*, iNOS, HIF-1*α*, COX-2, VEGF, and substance P levels were enhanced in the needling-treated muscle, accompanied by an increase in substance P in the DRG and a reduction in serum *β*-endorphin level; and (2) 5 d after the 5D treatment, these higher levels of TNF-*α*, iNOS, HIF-1*α*, COX-2, VEGF, and substance P were maintained, whereas the *β*-endorphin levels in the biceps femoris and serum increased. The 5D treatment of the biceps femoris with a taut band and MTrSs seemed to have a higher dosage than the 1D treatment; the 5D treatment may not have provided a suitable stimulus to activate *β*-endorphin and reduce the substance P level. TNF-*α* and COX-2 were implied to be involved in this possible damage of intensive dry needling. This result was supported by a study demonstrating that exercise-induced muscle damage was associated with an increase in COX-2 and TNF-*α* levels [39]. 

In this study, TNF-*α* overexpression was found in some traces of the needle penetrating into the biceps femoris. Although TNF-*α* levels in the biceps femoris and serum were enhanced by dry needling either in the 1D or the 5D treatment, abundant TNF-*α* accumulation and inflammatory cells were observed immediately and 5 d after the 5D treatment. The 5D treatment-activated COX-2 level was also higher than the 1D treatment in the biceps femoris. The increase in COX-2 expression was shown to be associated with the release of substance P, evoked by noxious stimuli from cultured DRG neurons [40]. A more likely possibility is that 5D is an overloaded invasive manipulation to cause excess damage in the skeletal muscle fibers, stimulate excessive noxious inputs, and increase the release of substance P. The result of this study indicates that pain level was raised after long-term dry needling. Our results also showed that *β*-endorphin increases in 5D group 5 days after dry needling, but not immediately after dry needling. This result could be supported by a study demonstrating that the *β*-endorphin messenger RNA expression was more predominate in the later phases of inflammation (peaking on day 14), leading to attenuated pain responses [41]. Whereas 5D increased the *β*-endorphin levels 5 d after ceasing treatment, the substance P levels in both the biceps femoris and the DRG did not decrease. This result could be supported by a human study demonstrating that an increased level of *β*-endorphin is insufficient to inhibit pain in the temporomandibular joint [42]. 

More studies showed that HIF-1*α* upregulation can be induced not only by hypoxic stress but also mechanical stress [43]. Induction of HIF target genes, including VEGF and iNOS, can promote angiogenesis, vasodilation, and altered glucose metabolism in hypoxic tissues [44, 45]. Studies using enhanced HIF-1*α* expression suggest that HIF-1*α* upregulation is a beneficial therapeutic modality for hypoxia/ischemia [45, 46]. The region containing numerous MTrPs can become focally ischemic because of limited oxygen supply by compression of the muscle contracture [2, 3]. Human studies also suggest that local, temporary hypoxia and blood flow reduction within muscle fibers in patients with trapezius myalgia, as well as the degree of hypoxia and impaired circulation, are correlated of pain intensity [47–49]. Our results also show that the 5D treatment can enhance HIF-1*α*, iNOS, and VEGF production in the needling-treated muscle. Thus, the increases in HIF-1*α* protein levels can upregulate VEGF protein expression, potentially increasing capillarity in the skeletal muscle. Therefore, the expression of HIF-1*α*, iNOS, and VEGF proteins can be key to improving circulation in muscles containing MTrSs after intensive dry needling. However, the long-term effects (>5 d) on the circulation after the 5D treatment remain unclear and need a long-term, follow-up study. 

In addition, a skeletal muscle is a dynamic tissue with an extraordinary capacity for repair after an injury [50]. TNF-*α*, iNOS, and VEGF are essential molecules involved in cellular events to activate the formation of new blood vessels and repair injured muscles in the process [50–52]. In this study, the 5D treatment enhanced the expression of these proteins, and thus, a 5D-induced muscle injury that can promote the rearrangement and repair of skeletal muscles with a taut band is of particular interest. Prevention of muscle fibrosis is the main objective of improving muscle healing following an injury from 5D. A further follow-up study must be conducted to investigate whether intensive trigger point dry needling of 5D affects the rearrangement of skeletal muscles with a taut band. 

### 4.3. Limitation of the Study

The primary disadvantage of this study is the lack of pain behavioral assessments to confirm the correlation between the alterations of biochemicals and the intensity of muscle pain after the 5D repetitive manipulation of dry needling in rabbits. Using our multiple quick insertion technique, we observed that long-term dry needling was much less effective compared with short-term dry needling in alleviating pain. We speculate that the sharp tip of the acupuncture needles causes additional muscle damage after the 5D treatment. A human study demonstrated that visual analog scales were not significantly decreased after six sessions of dry needling compared with the placebo group. The authors conclude that multiple insertions of dry needling with a blunt needle caused one type of local muscle injury, causing pain in the treatment of MPS [53]. In addition, dry needling using an acupuncture needle can cause even more severe damage compared with blunt needles [53]. Therefore, the 5D treatment using an acupuncture needle can increase the biochemicals associated with inflammation and pain because of excessive muscle damage, thus increasing the intensity of pain.

## 5. Conclusion

The hypothesis that dry needling at MTrSs can modulate biochemicals associated with pain, inflammation, and hypoxia depending on the dry needling dosage is supported by our data. The findings of this study can clarify the biochemical mechanisms induced by dry needling. This study can elucidate the analgesic action of dry needling in treating soft tissue systems and lead to the development of new therapeutic strategies to treat MPS. 

## Figures and Tables

**Table 1:** Summary of the biochemical effects in the bicep femoris, DRG, and serum affected by one- and five-dosage dry needling in the biceps femoris with MTrSs.

Biochemicals	Immediately after DN		5 days after DN	
	1D	5D	1D	5D
Biceps femoris				
*β*-endorphin	↑	—	—	↑
Substance P	↓	↑	—	↑
TNF-*α*	↑	↑↑	↑	↑↑
iNOS	—	↑	—	↑
HIF-1*α*	—	↑	—	↑
COX-2	—	↑	—	↑
VEGF	—	↑	—	↑
DRG				
Substance P	↓	↑	—	↑
Serum				
*β*-endorphin	↑	↓	—	↑
TNF-*α*	↑	↑↑	↑	↑↑

↑ indicates the significant increase in dry needling-treated groups compared with their sham groups. ↓ indicates reduction of Substance P in 1D group immediately after DN. ↑↑ indicates the significant increase in the 5D group compared with the 1D group. — indicates no significant difference between the dry needling-treated groups and the sham groups. Abbreviations: 1D: one-dosage dry needling; 5D: five-dosage dry needling; COX-2: cyclooxygenase-2; DN: dry needling; DRG: dorsal root ganglion; HIF-1*α*: hypoxia-inducible factor-1*α*; iNOS: inducible isoform of nitric oxide synthases; TNF-*α*: tumor necrosis factor-*α*; VEGF: vascular endothelial growth factor.
